# Case report: Durable response to ruxolitinib in a child with *TREX1*-related disorder

**DOI:** 10.3389/fped.2023.1178919

**Published:** 2023-04-28

**Authors:** Yasir Bin Khathlan, Sajdi Almutairi, Fahad B. Albadr, Abdullah A. Alangari, Abdulrahman Alsultan

**Affiliations:** ^1^ Department of Pediatrics, College of Medicine, King Saud University, Riyadh, Saudi Arabia; ^2^ Department of Radiology and Medical Imaging, King Saud University Medical City and College of Medicine, King Saud University, Riyadh, Saudi Arabia; ^3^ Oncology Center, King Saud University Medical City, Riyadh, Saudi Arabia

**Keywords:** *TREX1* gene, JAK—STAT signaling pathway, ruxolinitib, child, pancytopenia

## Abstract

**Background:**

JAK inhibitors are useful in treating interferonopathies, presumably because they downregulate the JAK/STAT signaling. There are limited studies about the safety and effectiveness of using JAK inhibitors in children with *TREX1*-related disorders.

**Case presentation:**

We report an 8-year-old female who presented at five years of age with features suggestive of hemophagocytic lymphohistiocytosis (HLH)-like disorder. The infectious disease workup was negative. Neurological assessment was normal. A brain CT scan was performed because of headache. It showed a faint subcortical calcification at right frontal lobe and almost symmetrical calcification within the basal ganglia. Brain MRI showed bilateral symmetrical globus pallidus, high T1 signal intensities, and a few scattered nonspecific FLAIR hyperintensities in subcortical and deep white matter. IVIG as an immune modulating agent was administered initially which led to the resolution of fever, improvement of blood count parameters, inflammatory markers, and normalization of liver enzymes. The child remained afebrile with no significant events for several months, then had disease flare up. The patient was started on pulse methylprednisolone 30 mg/kg for three days, then continued on 2 mg/kg. Whole exome sequencing revealed a novel heterozygous missense *TREX1* mutation NM_016381.3:c.223G > A p.(Glu75Lys). The child was started on ruxolitinib, 5 mg orally twice daily. The child has prolonged, durable remission after initiating ruxolitinib with no adverse effects. Steroids were tapered off and the patient is no longer on IVIG. The patient is still on ruxolitinib for more than two years.

**Conclusion:**

This case highlights the potential role of ruxolitinib in the treatment of *TREX1*-related disorders. A longer follow-up period is required to evaluate the long-term outcome.

## Background

Detrimental loss of function variants (autosomal recessive or dominant negative) in *TREX1* (three prime repair exonuclease 1) gene are associated with autoinflammatory disorders secondary to type I interferonopathies (1). The clinical phenotype is variable and includes Aicardi–Goutières syndrome (AGS), familial chilblain lupus (FCL), retinal vasculopathy with cerebral leukoencephalopathy and systemic manifestations (RVCLS), and SLE (2–4). JAK inhibitors are useful in treating interferonopathies, presumably because they downregulate the JAK/STAT signaling (5, 6). There are limited studies about the safety and effectiveness of using JAK inhibitors in children with *TREX1*-related disorders (7–9). We report a child who presented with features suggestive of hemophagocytic lymphohistiocytosis (HLH)-like disorder who was later diagnosed with *TREX1*-related disorder based on whole exome sequencing (WES) analysis. The child has prolonged, durable remission after initiating ruxolitinib with no adverse effects.

## Case presentation

Our patient is an 8-year-old female who presented at five years of age with two months history of fever, erythematous rash over the forehead, fatigue, and poor appetite. The parents are consanguineous, and two siblings have a history of seizure disorder in the presence of normal brain MRI. Weight was on the 5th percentile, and height was below the 5th percentile. Physical examination showed enlarged and tender lymph nodes in the right anterior cervical and right inguinal areas with no hepatosplenomegaly. CBC showed pancytopenia with WBC 2.1 × 10^9^/L, ANC 0.9 × 10^9^/L, ALC 1.1 ×10^9^/L, Hb 6.8 g/dl, MCV 80 fl (normal 77–85), and platelet 67 × 10^9^/L. The reticulocyte production index was 0.2%. A peripheral blood smear was significant for increased schistocytes, bilobulation of some neutrophils, few reactive lymphocytes, and occasional giant platelets. ESR was 17 mm/h, and CRP was 7 mg/L. PT and aPTT were normal. There was an increase in ALT (187 units/L, normal 20–65) and AST (318 units/L, normal 15–37). Otherwise, liver function tests were unremarkable. LDH was >1,000 units/L (normal 84–246). Fibrinogen was slightly low, 1.9 g/L (normal 2–4), and ferritin was >2,000 mcg/L. Triglycerides was 2.37 mmol/L (normal 0.34–1.13). The metabolic panel showed mild hypocalcemia 1.87 mmol/L (normal 2.1–2.55), hypophosphatemia 1.42 mmol/L (normal 1.45–1.78), normal urea and creatinine, and urine examination was normal. Immunoglobulin levels were normal, and quantitative lymphocyte subsets showed a slight decrease in B-cell 334/ul (normal 400–800/ul), and NK-cell 105/ul (normal 200–400/ul). ANA was 1:160 fine speckled with normal C3 and C4. Hemoglobin electrophoresis and G6PD level were normal. The infectious disease workup was negative which included negative blood and urine cultures, negative serology for CMV, EBV, HIV, HBV, and HCV, negative brucella culture, negative aspergillus galactomannan, and negative Quantiferon test for tuberculosis. Bone marrow examination showed hypocellular marrow with a marked increase in histiocytes, occasional hemophagocytic activity, and markedly reduced granulopoiesis. Echocardiogram was normal. Ultrasound abdomen showed mildly swollen kidneys with a mild increase in cortical echogenicity. The patient was diagnosed with hypothyroidism and was started on levothyroxine.

**Figure 1:**
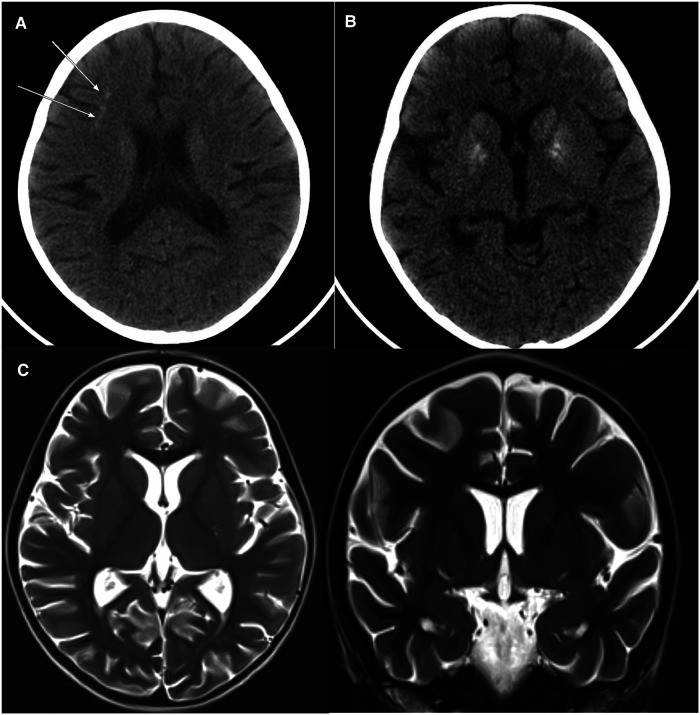
Axial non-contrast CT brain with soft tissue window: there is a faint subcortical calcification at the right frontal lobe (**A**) and almost symmetrical calcification within the basal ganglia (**B**). Axial and coronal TW2 Brain MRI: Mild generalized brain atrophy was noted. Gyration, myelination, and brain morphology are otherwise normal (**C**).

**Figure 2:**
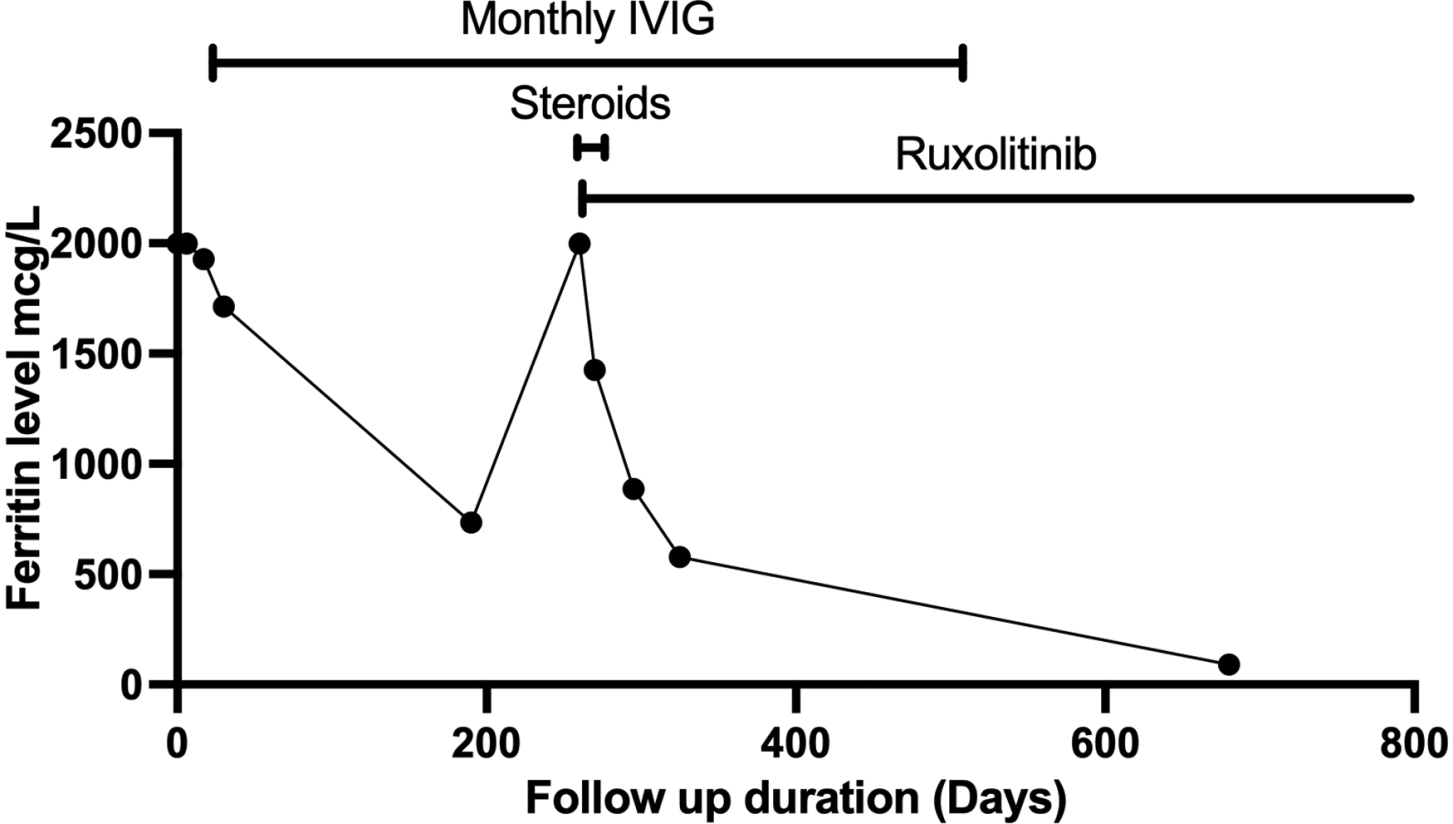
The trend in ferritin levels in response to different treatments that were used.

The fever persisted despite broad-spectrum antibiotics (piperacillin-tazobactam and amikacin) and anti-fungal therapy (amphotericin B). There were intermittent episodes of hypertension (maximum 138/84 stage II). Neurological assessment was normal. The ophthalmology exam was normal. A brain computerized tomography scan was performed because of headache. It showed a faint subcortical calcification at right frontal lobe and almost symmetrical calcification within the basal ganglia. Brain magnetic resonance imaging showed bilateral symmetrical globus pallidus, high T1 signal intensities, and a few scattered nonspecific fluid attenuated inversion recovery (FLAIR) hyperintensities in subcortical and deep white matter. There was mild generalized brain atrophy, Figure 1. The patient fulfilled the modified criteria of HLH 2009 but did not fulfill the diagnostic criteria for HLH-2004; only four criteria were present: fever, pancytopenia, Ferritin ≥ 500 mcg/L, and hemophagocytic activity in the bone marrow. However, there was no splenomegaly, triglycerides ≥3 mmol/L, or low fibrinogen. NK-cell activity and soluble CD25 were not assessed. Thus, HLH therapy was not started (10, 11). Nevertheless, immune dysregulation disorder was suspected, and we started IVIG (1 g/kg) as an immune modulating agent was administered starting on day 16 of admission and was repeated regularly every 3–4 weeks. This has led to the resolution of fever, improvement of blood count parameters and normalization of liver enzymes. However, the ferritin level remained relatively high at 735 mcg/L, Figure 2. CBC showed WBC 3.7 × 10^9^/L, ANC 1.5 × 10^9^/L, ALC 1.5 ×10^9^/L, Hb 9.9 g/dl, and platelet 306 × 10^9^/L. The reticulocyte count was 2.1%. ALT was (56 units/L) and AST (was 79 units/L). The patient was discharged in stable condition with a plan to administer IVIG monthly. The child remained afebrile with no significant events for six months, then was again admitted with fever (38–39°C), oral ulcers, pancytopenia, ferritin >2,000 mcg/L, LDH >1,000 unit/L, ALT 147 units/L, AST 508 units/L, and normal fibrinogen. The infectious disease workup was again negative. The patient was started on pulse methylprednisolone 30 mg/kg for three days given the concerns of secondary HLH due to autoinflammatory disorder, then continued on 2 mg/kg. Subsequently, whole exome sequencing (WES) was requested given the concern of inherited immune dysregulation disorders or HLH which revealed a novel heterozygous missense *TREX1* mutation NM_016381.3:c.223G > A p.(Glu75Lys) which is located in N-terminal catalytic domain and this amino acid change is predicted to impact the protein stability (12). This variant was not reported in the genome aggregation database, exome sequencing project, and 1,000 genome project. *In silico* prediction was performed to assess the effect of this variant; it is predicted to be pathogenic by Align-GVGD *C*55 and disease-causing by MutationTaster. Thus, a diagnosis of autosomal dominant *TREX1*-related disorder such as AGS was considered, and the patient was started on ruxolitinib, 5 mg orally twice daily as an emerging treatment option for interferonopathies. Steroids were tapered off, and the patient is no longer on IVIG for more than one year, Figure 2. The patient is still on ruxolitinib for more than two years. Most recent blood work while on ruxolitinib showed CBC: WBC 4.7 × 10^9^/L, ANC 1.8 × 10^9^/L, ALC 2.3 ×10^9^/L, Hb 12.8 g/dl, and platelet 438 × 10^9^/L. Ferritin 90 mcg/L. Interferon signature was not measured. During treatment with ruxolitinib, no signs of autoinflammation were observed. The treatment was well tolerated, with no serious side effects. Segregation analysis of the family was not performed based on parental preference.

## Discussion and conclusions

Although our patient exhibited characteristics similar to those of HLH based on modified HLH 2009 criteria but did not fulfill the HLH-2004 criteria as there was no splenomegaly, hypofibrinogenemia, or hypertriglyceridemia (10, 11). HLH-2004 criteria have limitations for diagnosing HLH disease mimics (secondary HLH) (13, 14). Autoinflammatory illnesses related to AGS are associated with interferon signatures and present with HLH-like features such as organomegaly, cytopenias, and fever. Developmental delay, microcephaly, cardiomyopathy, hypothyroidism, myositis, arthritis, and retinopathy are other characteristics that can be seen in AGS patients. A brain MRI may reveal signs of moyamoya disease, cerebral atrophy, cerebral calcifications, or leukoencephalopathy (15).

Our patient initially experienced a transient response to IVIG. However, the patient developed disease exacerbations that responded effectively to the JAK1/2 inhibitor, ruxolitinib. One case of FCL with a *TREX1* mutation that responded well to ruxolitinib was reported (8). Patients with AGS, including those with *TREX1* mutations, who were treated with baricitinib, a JAK1/2 inhibitor, experienced significant clinical improvement (9). Tofacitinib, a pan-JAK inhibitor, produced remission in a patient with FCL associated with *TREX1* mutation, despite the patient's need for occasional steroid treatment for disease flare-ups, Supplementary Table S1 (7). In disorders associated with type I interferonopathies, the choice of JAK inhibitor, appropriate dose, and duration have yet to be established. Ruxolitinib is only licensed for children 12 years and older who have steroid-refractory acute graft vs. host disease (GVHD) or chronic GVHD after at least one line of systemic therapy has failed (16, 17). The recommended dose in children is not well defined, the REACH4 study proposed the following dosing of ruxolitinib based on age in children with acute GVHD; 4 mg/m^2^ BID ≥2years to <6years, 5 mg BID ≥6years to <12years, and 10 mg BID ≥12years to <18years (18). Close monitoring of CBC is required because cytopenias are the most frequent adverse events, followed by infections.

It is believed that cytosolic DNA accumulation is the cause of type I interferonopathies in patients with TREX1-related disorder (6). JAK inhibitors such as ruxolitinib or baricitinib inhibit STAT phosphorylation and decrease IFN response gene score in patients with interferonopathies (5, 19, 20). Recent studies of how ruxolitinib works in murine models of primary and secondary HLH showed that ruxolitinib suppresses the production of proinflammatory cytokines, including IFN-*γ*, IL-6, IL-12, GM-CSF, and TNF-α, as well as the proliferation and activation of T-cells, monocytes, and neutrophils in inflamed organs (21–23).

In conclusion, patients with HLH-like characteristics should be evaluated for autoinflammatory disorders, such as *TREX1*-related disorders. This case highlights the potential role of ruxolitinib in the treatment of *TREX1*-related disorders. A longer follow-up period is required to evaluate the long-term outcome.

## Data Availability

The original contributions presented in the study are included in the article/supplementary material, further inquiries can be directed to the corresponding author.
