# Supplementary material for: Case report: Durable response to ruxolitinib in a child with TREX1-related disorder
Source: Front Pediatr. 2023 Apr 28;11:1178919. doi: 10.3389/fped.2023.1178919 (PMC10175768; doi:10.3389/fped.2023.1178919)
Supplement: Supplementary file 1 [file Table1.pdf]

Supplemental Table 1. Clinical and molecular characteristics of children with *TREX1*-related disorders treated with JAK inhibitors

**Features**	**Our patient**	**Zhang et al. (7)**	**Briand et al. (8)**	**Vanderver et al. (9)**
**Number of patients**	1	1	1	5
**Gender (male/female)**	Female	Female	Female	na
**Ethnicity**	Arab	Chinese	na	na
**Age of onset (years)**	5	2	0.5	Median 0.5
**Phenotype**	HLH-like<br>Mild AGS	FCL	FCL	AGS
***TREX1* mutation**	Heterozygous<br>c.223G>A p.Glu75Lys	Compound heterozygous<br>c. 227C>T p.Ala76Val<br>c.458dupA p.Gln153fs3	Heterozygous<br>c.52G>A p.Asp18Asn	Compound heterozygous<br>c.341G>A p.Arg114His<br>c.667G>A p.Ala223Thr (n=2)<br>Compound heterozygous<br>c.341G>A p.Arg114His<br>c.416delC p.Ala139ValfX21 (n=1)<br>Compound heterozygous<br>c.341G>A p.Arg114His<br>c.907A>C p.Thr303Pro (n=1)<br>Homozygous<br>c.341G>A p.Arg114His (n=1)
**Treatment**	IVIG<br>Steroids<br>Ruxolitinib	Tofacitinib<br>Steroids during winter	Steroids<br>Hydroxychloroquine<br>Ruxolitinib	Baricitinib
**Outcome**	Alive in complete remission after starting ruxolitinib	Alive with marked improvement after starting Tofacitinib	Alive with near complete remission after starting ruxolitinib	Significant clinical improvement
**Follow up duration (years)**	3	2	3	1
